# Geriatric Nutritional Risk Index is a risk factor for long‐term decreases in patient‐reported outcome measures following total knee arthroplasty

**DOI:** 10.1002/jeo2.70170

**Published:** 2025-02-12

**Authors:** Yasuhiko Kokubu, Shinya Kawahara, Satoshi Hamai, Yukio Akasaki, Taishi Sato, Toshiki Konishi, Takahiro Inoue, Yasuharu Nakashima

**Affiliations:** ^1^ Department of Orthopaedic Surgery, Graduate School of Medical Sciences Kyushu University Fukuoka Japan

**Keywords:** geriatric, Geriatric Nutritional Risk Index, patient‐reported outcome measures, preoperative malnutrition, total knee arthroplasty

## Abstract

**Purpose:**

Total knee arthroplasty (TKA) is an effective treatment for alleviating pain and improving function in patients with end‐stage knee osteoarthritis. However, factors influencing long‐term patient‐reported outcome measures (PROMs) remain underexplored. This study aimed to evaluate the relationship between preoperative nutritional status, specifically the Geriatric Nutritional Risk Index (GNRI), and the long‐term decline in PROMs following TKA.

**Methods:**

We conducted a retrospective cohort study including patients who underwent TKA between 2000 and 2009. PROMs were assessed using the Knee Society Score (KSS) at two time points: an initial evaluation in 2012 (median postoperative 4 years) and a follow‐up in 2023 (median 13 years). Preoperative GNRI, body mass index (BMI), and other demographic and clinical data were collected from medical records. Statistical analysis included paired *t*‐tests and multivariate logistic regression to identify independent risk factors for long‐term decline in KSS scores.

**Results:**

A total of 75 patients completed follow‐up assessments. Over the 11‐year follow‐up period, there was a significant decrease in the KSS functional activity scores (*p* < 0.001), with 47 patients experiencing a decline exceeding the minimal clinically important difference. A multivariate analysis revealed low preoperative GNRI (*p* = 0.0043) as a significant risk factor for long‐term decline in PROMs.

**Conclusion:**

Preoperative malnutrition, as indicated by a low GNRI, is a significant risk factor for long‐term decline in functional outcomes following TKA. These findings highlight the importance of preoperative nutritional interventions and rehabilitation for improving the long‐term outcomes of patients undergoing TKA.

**Level of Evidence:**

Level III, retrospective cohort study.

**Abbreviations**
ASA: American Society of Anesthesiologists
BMI: body mass index
GNRI: Geriatric Nutritional Risk Index
KSS: Knee Society Score
MCID: minimal clinically important difference
PROMs: patient‐reported outcome measures
TKA: total knee arthroplasty

## INTRODUCTION

Total knee arthroplasty (TKA) is an effective treatment for pain reduction and physical disability in older patients with end‐stage knee osteoarthritis. With the extension of life expectancy and improvements in component technology, long‐term survival with favourable knee function is expected after TKA. In recent years, many studies have investigated the factors that contribute to good postoperative outcomes using patient‐reported outcome measures (PROMs) [10, 15]. Although long‐term PROMs following TKA have often been reported [7, 17], few studies have investigated longitudinal changes over a longer follow‐up period.

Although infrequent, fatal complications or the need for revision surgery occurs in the long term after arthroplasty in some cases. Poor preoperative nutritional status in older patients has been increasingly recognized to be significantly associated with higher rates of postoperative complications and mortality [3, 6, 9]. The Geriatric Nutritional Risk Index (GNRI), calculated from current body weight, ideal body weight, and serum albumin levels, is a tool used to screen nutritional status and has been validated as a predictor of complications and mortality following arthroplasty [2, 5, 8, 23]. However, no reports have investigated the relationship between the GNRI and long‐term postoperative PROMs after TKA.

This study aimed to investigate the factors influencing the longitudinal changes in PROMs over a long follow‐up period after TKA. We hypothesized that poor preoperative nutritional status is a risk factor for long‐term deterioration of PROMs.

## METHODS

### Patient selection

**Figure 1:**
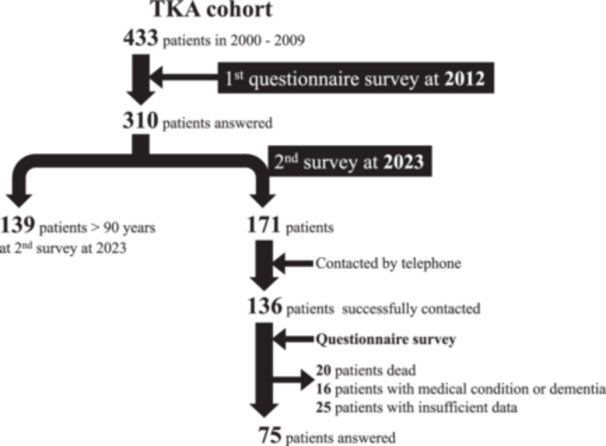
Strengthening the Reporting of Observational Studies in Epidemiology diagram for the inclusion process.

Patients diagnosed with osteoarthritis who underwent TKA between February 2000 and December 2009 were included in this study. At our hospital, post‐operative patients are followed up at 1 month, 3 months, 6 months, 1 year, and 2 years after surgery following our routine follow‐up protocol. After 2 years, patients are followed up every other year; however, those having difficulty in visiting the hospital due to distance or other reasons are transferred to a clinic with easier access. Therefore, at the time of the initial assessment in 2012, soon after the Knee Society Score (KSS 2011) was introduced, scores were obtained through mailed questionnaires, and the data were obtained from a cross‐sectional perspective with a retrieval rate of 72%. All patients who responded were aged ≤90 at the time of the survey. The mean postoperative follow‐up duration was 5.5 years. A second assessment using the KSS 2011 was conducted in April 2023. At the time of the second assessment, patients >90 years were excluded because they were considered more likely to have difficulty in understanding, completing, and returning the questionnaire [13, 20]. The health status of all patients, including postoperative complications and mortality, was verified through telephone interviews using contact information recorded in electronic medical records. Thirty‐five patients could not be reached via the telephone numbers listed in the electronic medical record, but 136 were successfully contacted. No difference between those who were contacted and those who could not be contacted was observed in descriptive data or initial survey results (Appendix S1: Table 1). Furthermore, 20 and 16 patients were unable to return the questionnaires due to death and advanced dementia or medical conditions, respectively. Twenty‐five patients with insufficient preoperative blood test data, comorbidities, or postoperative imaging were excluded. Finally, 75 patients consented to a second survey using the KSS 2011, and all returned the questionnaires and provided informed consent before participation (Figure 1). Demographic data obtained from electronic medical records included age, sex, body mass index (BMI), preoperative comorbidities, and the American Society of Anesthesiologists (ASA) classification at the time of surgery. Serum albumin levels were reviewed from the preoperative blood test data. Surgical details such as component design (posterior‐stabilized [PS] or cruciate‐retaining [CR]), use of navigation systems, operating time, and intraoperative blood loss were investigated using the electronic medical record. Radiographic data, including the hip–knee–ankle angle, were measured using standing full‐leg radiographs obtained preoperatively and postoperatively to assess the similarity of baseline alignments of the knees. Measurements were repeated twice by one examiner (Y. K.) and once by another examiner (S. K.) in the study group. The intra‐ and inter‐class correlation coefficients for the measurements were good (0.91 and 0.88, respectively). The study procedures were approved by the local institutional review board (number: 2020‐204) and conducted according to the 1964 Declaration of Helsinki. All included patients provided informed consent.

### Statistical analyses

Univariate analysis was used to identify parameters that affected the results of the first (2012) cross‐sectional survey. Parameters with *p* values < 0.2 were included in a multivariable model. Paired *t* tests were used to compare the results of the first (2012) and second longitudinal surveys (2023). Additional analyses were performed for the subcategories that showed significant differences (KSS functional activity score). Based on previous studies, the minimal clinically important difference (MCID; 10.0 points in total, 4.1 points in functional activities) in the KSS score was used as a reference values [12, 19, 22]. The patients were categorized into two groups, including those whose scores worsened beyond the MCID threshold (decrease greater than the MCID) and controls. The parameters of each group were compared, and variables with *p* values < 0.2 were included in a multivariable model to identify the independent risk factors for long‐term deterioration in PROMs. A multivariate logistic regression analysis was performed using the stepwise variable‐entry method. Because the GNRI calculation depends on BMI and albumin level, only the GNRI was selected as a variable to avoid confounding. Continuous variables are presented as mean and standard deviation. Statistical analyses were performed using JMP version 15.0 (SAS Institute).

## RESULTS

Details of the included patients are presented in Tables 1a and 1b. Of the 310 patients included in the initial (2012) survey, 46 (15%) exhibited malnutrition, and 14 patients underwent additional surgery (5 cases of infection, 5 cases of aseptic loosening, and 4 cases of periprosthetic fracture). Of the 75 people included in the second (2023) survey, 10 (13%) exhibited malnutrition, and 9 patients underwent additional surgery (1 case of infection, 5 cases of aseptic loosening, and 3 cases of periprosthetic fracture). The KSS of the first and second cross‐sectional surveys are shown in Tables 2a and 2b, and those of longitudinal surveys in Table 2c. In the first cross‐sectional study, age at surgery (*p* < 0.001) and malnutrition (*p* = 0.0043) were identified as significant factors that decreased KSS (Tables 3a and 3b). In the second cross‐sectional study, age at surgery (*p* = 0.0372), malnutrition (*p* = 0.0014), and additional surgery (*p* < 0.001) were identified as significant factors that decreased KSS (Tables 4a and 4b).

**Table 1a:** Descriptive data and radiographic data of the first survey cohort.

Parameters	*n* = 310
Age at surgery (year)	74 (69, 79)
Sex, male, female, *n* (%)	48 (15), 262 (85)
BMI (kg/m^2^)	25.5 (22.8, 27.9)
Preoperative albumin (g/dL)	4.2 (4.0, 4.4)
Preoperative GNRI score	110.4 (103.9, 116.7)
Preoperative GNRI < 98, *n* (%)	46 (15)
ASA class 1, 2, 3; *n* (%)	1: 18 (6), 2: 246 (79), 3: 46 (15)
Operation time (min)	103 (87, 123)
Bleeding (mL)	36 (10, 45)
Component design, CR, PS, *n* (%)	74 (24), 236 (76)
Use of navigation, *n* (%)	53 (17)
Preoperative HKA angle (°)	9.0 (0.3, 14.0)
Postoperative HKA angle (°)	−0.7 (−3, 2)
Additional surgery, *n* (%)	14 (5)
Follow‐up period (year)	4 (2, 6)

*Note*: Data are expressed as median and interquartile range.

Abbreviations: ASA, American Society of Anaesthesiologists; BMI, body mass index; CR, cruciate‐retaining; GNRI, Geriatric Nutritional Risk Index; HKA, hip‐knee‐ankle; PS, posterior‐stabilized.

**Table 1b:** Descriptive data and radiographic data of the second survey cohort.

Parameters	*n* = 75
Age at surgery (year)	68 (61, 72)
Sex, male, female, *n* (%)	13 (19), 62 (81)
BMI (kg/m^2^)	26.9 (22.9, 29.8)
Preoperative albumin (g/dL)	4.2 (4.1, 4.4)
Preoperative GNRI score	112.5 (104.8, 120.5)
Preoperative GNRI < 98, *n* (%)	10 (13)
ASA class 1, 2, 3; *n* (%)	1: 3(4), 2: 61(81), 3:11(15)
Operation time (min)	109 (91, 129)
Bleeding (mL)	33 (10, 50)
Component design, CR, PS, *n* (%)	16 (21), 59 (79)
Use of navigation, *n* (%)	13 (18)
Preoperative HKA angle (°)	10.5 (0.3, 13.0)
Postoperative HKA angle (°)	−0.5 (−4, 2)
Additional surgery, *n* (%)	9 (12)
Follow‐up period (year)	13 (12, 16)

*Note*: Data are expressed as median and interquartile range.

Abbreviations: ASA, American Society of Anaesthesiologists; BMI, body mass index; CR, cruciate‐retaining; GNRI, Geriatric Nutritional Risk Index; HKA, hip‐knee‐ankle; PS, posterior‐stabilized.

**Table 2a:** Knee Society Score 2011 scores of the first survey cohort.

Parameters	Survey at 2012
Knee Society Score 2011	103 (83, 129)
Symptom (0–25)	20 (14, 23)
Satisfaction (0–40)	22 (18, 30)
Expectation (0–15)	9 (8, 11)
Functional activity (0–100)	53 (36, 70)

*Note*: Data are expressed as median and interquartile range.

**Table 2b:** Knee Society Score 2011 scores of the second survey cohort.

Parameters	Survey at 2012
Knee Society Score 2011	99 (74, 132)
Symptom (0–25)	20 (13, 24)
Satisfaction (0–40)	24 (18, 30)
Expectation (0–15)	9 (9, 12)
Functional activity (0–100)	47 (35, 72)

*Note*: Data are expressed as median and interquartile range.

**Table 2c:** Knee Society Score 2011 scores of the second survey cohort at two assessment points.

Parameters	Survey at 2012	Survey at 2023	*p* value
Knee Society Score 2011	117 (90, 138)	99 (74, 132)	0.0059
Symptom (0–25)	20 (15, 23)	20 (13, 24)	0.1853
Satisfaction (0–40)	24 (18, 30)	24 (18, 30)	0.9617
Expectation (0–15)	9 (9, 11)	9 (9, 12)	0.5014
Functional activity (0 to 100)	61 (43, 80)	47 (35, 72)	<0.001

*Note*: Data are expressed as median and interquartile range.

**Table 3a:** Univariate analysis of KSS2011 scores of the first survey cohort.

Parameters	*p* value
Age at surgery (year)	0.0005
Female	0.0971
BMI (kg/m^2^)	0.1162
Preoperative albumin (g/dL)	0.0004
Preoperative GNRI score	0.0028
Preoperative GNRI < 98	0.0357
ASA class	0.2451
Operation time (min)	0.9608
Bleeding (mL)	0.4435
Component design: CR	0.2121
Use of navigation	0.1392
Preoperative HKA angle (°)	0.8856
Postoperative HKA angle (°)	0.7756
Additional surgery	0.6357
Follow‐up period (year)	0.0933

Abbreviations: ASA, American Society of Anaesthesiologists; BMI, body mass index; CR, cruciate‐retaining; GNRI, Geriatric Nutritional Risk Index; HKA, hip‐knee‐ankle; KSS, Knee Society Score.

**Table 3b:** A multivariate logistic regression analysis to identify the risk factor for KSS total score of the first survey.

Parameters	*β* value (95% CI)	*p* value
Age at surgery	−3.84 (−1.42 to −0.46)	0.0002
Female	0.73 (−8.42 to 18.07)	0.4744
Preoperative GNRI < 98	−7.26 (−12.49 to −2.04)	0.0043
Use of navigation	−1.62 (−9.00 to 0.89)	0.1074
Follow‐up period	−1.87 (−2.62 to 0.07)	0.0624

*Note*: The variables with *p* values of <0.2 in the univariate analysis were included in a multivariable model. A multivariate logistic regression analysis was performed using the stepwise variable entry method.

Abbreviations: CI, confidence interval; GNRI, Geriatric Nutritional Risk Index; KSS, Knee Society Score.

**Table 4a:** Univariate analysis of KSS2011 scores of the second survey cohort.

Parameters	*p* value
Age at surgery (year)	0.1021
Female	0.0138
BMI (kg/m^2^)	0.3318
Preoperative albumin (g/dL)	0.0385
Preoperative GNRI score	0.9489
Preoperative GNRI < 98	0.0214
ASA class	0.2587
Operation time (min)	0.4494
Bleeding (mL)	0.8231
Component design: CR	0.7605
Use of navigation	0.8060
Preoperative HKA angle (°)	0.2856
Postoperative HKA angle (°)	0.2752
Additional surgery	0.0159
Follow‐up period (year)	0.6052

Abbreviations: ASA, American Society of Anaesthesiologists; BMI, body mass index; CR, cruciate‐retaining; GNRI, Geriatric Nutritional Risk Index; HKA, hip‐knee‐ankle; KSS, Knee Society Score.

**Table 4b:** A multivariate logistic regression analysis to identify the risk factor for KSS total score of the second survey.

Parameters	*β* value (95%CI)	*p* value
Age at surgery	−1.13 (−2.34 to −0.07)	0.0372
Female	7.72 (−3.18 to 27.44)	0.2241
Preoperative GNRI < 98	−20.09 (−32.09 to −8.08)	0.0014
Additional surgery	−23.92 (−36.54 to −11.32)	0.0003

*Note*: The variables with *p* values of <0.2 in the univariate analysis were included in a multivariable model. A multivariate logistic regression analysis was performed using the stepwise variable entry method.

Abbreviations: GNRI, Geriatric Nutritional Risk Index; KSS, Knee Society Score.

The second longitudinal survey revealed a significant decrease in the total score (*p* < 0.001; Table 2c). A total of 33 patients experienced a decrease that exceeded the MCID threshold in the total score (Table 5a). Although the subcategories of symptoms, satisfaction, and expectations remained relatively stable, the functional activity scores significantly decreased (*p* < 0.001). A total of 47 patients experienced decreased function that exceeded the MCID threshold (Table 5b). Multivariate analysis revealed preoperative malnutrition as a significant risk factor for a long‐term longitudinal decrease in KSS‐total score (*p* = 0.0043; Table 6a) and KSS‐functional activity score (*p* = 0.0229; Table 6b). Upon categorizing patients according to nutritional status, those with malnutrition exhibited a significant long‐term longitudinal decline in KSS (Appendix S1: Table 2).

**Table 5a:** Comparison of parameters between patients with KSS total score decreased > MCID values (10 points) and control group in the second survey.

Parameters	Control (*n* = 42)	Decrease > MCID: 10 (*n* = 33)	*p* value
Change in KSS total	10 (0, 24)	−54 (−54, −20)	<0.0001
Age at surgery (year)	67 (59, 70)	68 (62, 74)	0.1779
Sex, male, female, *n* (%)	12 (29), 30 (71)	1 (3), 32 (97)	0.0045
BMI (kg/m^2^)	26.5 (22.8, 29.9)	27.5 (22.7, 30.8)	0.5219
Preoperative albumin (g/dL)	4.2 (4.1, 4.4)	4.2 (4.0, 4.4)	0.8760
Preoperative GNRI score	111.7 (104.6, 120.1)	116.5 (105.1, 121.3)	0.6655
Preoperative GNRI < 98, *n* (%)	3 (7)	7 (21)	0.0952
ASA class 1, 2, 3, *n* (%)	1: 2 (5), 2: 33 (78), 3: 7 (17)	1: 1 (3), 2: 28 (85), 3: 4 (12)	0.7833
Operation time (min)	114 (90, 131)	104 (93, 127)	0.8200
Bleeding (mL)	33 (12, 53)	33 (10, 48)	0.5006
Component design, CR, PS, *n* (%)	11 (27), 30 (73)	5 (15), 28 (85)	0.2667
Use of navigation, *n* (%)	6 (15)	7 (21)	0.5454
Preoperative HKA angle (°)	10.0 (8.4, 13.1)	11.0 (9.0, 14.0)	0.4841
Postoperative HKA angle (°)	−1.4 (−3.4, 1.6)	−0.3 (−4.3, 2.3)	0.5244
Additional surgery, *n* (%)	5 (12)	4 (12)	1.0
Follow‐up period (year)	13.9 (12.6, 16.3)	12.9 (11.6, 15.4)	0.2392

*Note*: Data are expressed as median and interquartile range.

Abbreviations: ASA, American Society of Anaesthesiologists; BMI, body mass index; CR, cruciate‐retaining; GNRI, Geriatric Nutritional Risk Index; HKA, hip‐knee‐ankle; KSS, Knee Society Score; MCID, minimal clinically important difference; PS, posterior‐stabilized.

**Table 5b:** Comparison of parameters between patients with KSS functional activity score decreased > MCID values (4.1 points) and control group in the second survey.

Parameters	Control (n = 28)	Decrease > MCID: 4.1 (n = 47)	*p* value
Change in KSS function	11 (2, 19)	−13 (−35, −10)	<0.0001
Age at surgery (year)	65 (59. 71)	68 (64, 72)	0.1490
Sex, male, female, *n* (%)	8 (29), 20 (71)	5 (11), 42 (89)	0.0619
BMI (kg/m^2^)	25.1 (21.3, 28.5)	28.0 (23.0, 31.4)	0.0374
Preoperative albumin (g/dL)	4.2 (4.1, 4.4)	4.2 (4.0, 4.4)	0.6868
Preoperative GNRI score	110.4 (105.0, 115.9)	117.5 (104.8, 122.0)	0.0825
Preoperative GNRI < 98, *n* (%)	1 (4)	8 (17)	0.1410
ASA class 1, 2, 3; *n* (%)	1: 2 (7), 2: 21 (75), 3: 5 (18)	1: 1 (2), 2: 40 (85), 3: 6 (13)	0.4418
Operation time (min)	113 (92, 132)	105 (91, 125)	0.6936
Bleeding (ml)	30 (10, 50)	33 (11, 54)	0.7603
Component design, CR, PS, *n* (%)	6 (21), 22 (79)	10 (21), 37 (79)	1.0
Use of navigation, *n* (%)	5 (18)	8 (17)	1.0
Postoperative HKA angle (°)	−1.0 (−3.5, 2.0)	−0.6 (−4.0, 2.0)	0.9713
Additional surgery, *n* (%)	2 (7)	7 (15)	0.4699
Follow‐up period (year)	13 (12 16)	13 (12, 16)	0.9738

*Note*: Data are expressed as median and interquartile range.

Abbreviations: ASA, American Society of Anaesthesiologists; BMI, body mass index; CR, cruciate‐retaining; GNRI, Geriatric Nutritional Risk Index; HKA, hip‐knee‐ankle; KSS, Knee Society Score; MCID, minimal clinically important difference; PS, posterior‐stabilized.

**Table 6a:** A multivariate logistic regression analysis to identify the risk factor for long‐term deterioration in KSS total score.

Parameters	*β* value (95% CI)	*p* value
Age at surgery	0.06 (−0.01 to 0.13)	0.1014
Preoperative GNRI < 98	1.57 (0.60 to 2.82)	0.0043

*Note*: The parameters of each group were compared, and variables with *p*‐values of <0.2 were included in a multivariable model. A multivariate logistic regression analysis was performed using the stepwise variable entry method.

Abbreviations: CI, confidence interval; GNRI, geriatric nutritional risk index; KSS, Knee society score; MCID, minimal clinically important difference.

**Table 6b:** A multivariate logistic regression analysis to identify the risk factor for long‐term deterioration in KSS functional activity score.

Parameters	*β*‐value (95% CI)	*p* value
Female	0.48 (−0.22 to 1.17)	0.1778
Preoperative GNRI < 98	1.27 (0.42 to 2.74)	0.0229

*Note*: The parameters of each group were compared, and variables with *p*‐values of <0.2 were included in a multivariable model. A multivariate logistic regression analysis was performed using the stepwise variable entry method.

Abbreviations: CI, confidence interval; GNRI, geriatric nutritional risk index; KSS, Knee society score.

## DISCUSSION

The present study evaluated the KSS 2011 at two time points over an 11‐year period, making it one of the longest to observe longitudinal changes using the KSS 2011. The long follow‐up period is a major strength of the present study. This study showed a significant longitudinal decrease in PROMs over time. The significant decrease, especially in functional activity score, likely reflects the natural age‐related decline in physical abilities, such as muscle strength and activity levels [18, 26]. Age at surgery was identified as a risk factor for unfavourable KSS in the cross‐sectional study at two time points. Previous reports have shown that surgery in older patients is associated with poor functional outcomes, which may explain the results of this cross‐sectional study [11, 21]. In addition, preoperative malnutrition, as indicated by a low GNRI score, was identified as a risk factor for unfavourable KSS not only in the cross‐sectional survey but also in the longitudinal survey.

In this study, the prevalence of malnutrition (defined as a GNRI score <98) was 15%, which is consistent with previous reports [6, 10]. The GNRI is increasingly used as an effective predictive tool for assessing nutritional status, incorporating easily obtainable measurements such as height, weight, and serum albumin level, which mitigate the influence of hepatic and renal impairment and fluid balance on nutritional status assessments [6, 9]. Recently, a low preoperative GNRI score has been reported to be associated with mortality, rehospitalization, and complications such as deep vein thrombosis and surgical site infection after joint arthroplasty surgery [5, 23]. In this study, the 35 patients who missed the second survey due to death or medical disease showed malnutrition compared to the 75 patients included in the second survey (Appendix S1: Table 3). In contrast, few studies have reported an association between postoperative PROMs and a low GNRI score. Guanzhi et al. reported that patients undergoing robot‐assisted TKA with low GNRI scores showed significantly greater improvement in KSS at 12 weeks postoperatively than those who underwent conventional TKA [14], and they highlighted the importance of patient selection given the costs associated with robot‐assisted TKA. Our study supports their findings from a long‐term perspective.

Malnutrition is closely associated with sarcopenia, which leads to poor postoperative outcomes, including poor immune function, prolonged hospitalization, and increased mortality due to muscle dysfunction [1, 3, 24]. The present study supports these reports in terms of PROMs. Many recent studies have reported the effectiveness of nutritional interventions such as optimizing nutritional status before surgery, avoiding preoperative fasting, postoperative use of high‐protein supplements, and early postoperative oral intake and muscle training in older individuals with frailty and sarcopenia [16, 25, 27, 28]. Preoperative nutritional status should be considered to create a comprehensive treatment plan including physical rehabilitation and nutritional interventions.

This study has several limitations. Given the retrospective nature of this study, the potential impact of these selection biases must be carefully considered. We used KSS 2011 as a postoperative PROM; however, preoperative assessment was not available. The first cross‐sectional study was conducted in 2012, after the introduction of the KSS 2011. Therefore, changes from preoperative values could not be evaluated. Other PROMs, such as Western Ontario and McMaster Universities Arthritis Index, Short Form‐36, and EQ‐5D, could have provided a more comprehensive understanding of the postoperative functional outcomes [4, 6, 29]. Furthermore, while the long follow‐up period is a strength of this study, there were variations in follow‐up timing. Therefore, considering these variations, we also investigated the follow‐up period in each sub‐analysis, but no significant differences were found. However, as indicated by the results of the longitudinal survey, PROMs tend to decrease as the post‐operative course progresses. Therefore, a long post‐operative follow‐up period may be a risk factor, especially for patients with malnutrition.

## CONCLUSION

Preoperative malnutrition may be a risk factor for the long‐term postoperative decline in PROMs. To achieve favourable long‐term functional outcomes in patients with preoperative malnutrition, creating a comprehensive treatment plan that includes physical rehabilitation and nutritional interventions is important.

## AUTHOR CONTRIBUTIONS

*Substantial contributions to research design, or the acquisition, analysis or interpretation of data*: Yasuhiko Kokubu, Shinya Kawahara, Satoshi Hamai, Yukio Akasaki, Taishi Sato, Toshiki Konishi, Takahiro Inoue, Yasuharu Nakashima. Drafting the paper or revising it critically: Yasuhiko Kokubu, Shinya Kawahara. Approval of the submitted and final versions: Yasuhiko Kokubu, Shinya Kawahara, Satoshi Hamai, Yukio Akasaki, Taishi Sato, Toshiki Konishi, Takahiro Inoue, Yasuharu Nakashima. All authors have read and approved the final submitted manuscript.

## CONFLICT OF INTEREST STATEMENT

The authors declare no conflicts of interest.

## ETHICS STATEMENT

The study procedures were approved by the local institutional review board (no. 2020‐204) and conducted in accordance with the 1964 Helsinki Declaration. All patients provided informed consent before participation.

## Supporting information

Supporting information.

## Data Availability

The data that support the findings of this study are available from the corresponding author, S. K., upon reasonable request.
