# Supplementary material for: Geriatric Nutritional Risk Index is a risk factor for long‐term decreases in patient‐reported outcome measures following total knee arthroplasty
Source: J Exp Orthop. 2025 Feb 12;12(1):e70170. doi: 10.1002/jeo2.70170 (PMC11815201; doi:10.1002/jeo2.70170)
Supplement: Supplementary file 1 — Supporting information. [file JEO2-12-e70170-s001.docx]

**Appendix Table 1**. Descriptive data on cases contacted and uncontacted at the time of the second survey.

| Parameters | Contacted (n = 136) | Uncontactable (n=35) | p-value |
| --- | --- | --- | --- |
| Age at surgery (year) | 70 (64, 74) | 69 (64, 73) | 0.3789 |
| Sex, Male, Female, n (%) | 29 (21), 107 (79) | 6 (17), 29 (83) | 0.6475 |
| BMI (kg/m^2^) | 26.1 (22.9, 29.1) | 26.9 (24.8, 29.3) | 0.2959 |
| Preoperative albumin (g/dl) | 4.2 (4.0, 4.4) | 4.3 (4.0, 4.4) | 0.1214 |
| Preoperative GNRI score | 111.5 (103.5, 119.7) | 116.2 (107.4, 121.2) | 0.1252 |
| Preoperative GNRI < 98, n (%) | 24 (17) | 2 (9) | 0.2972 |
| ASA class 1, 2, 3; n (%) | 1:6(4),2:105(77),3:25(19) | 1:2(6), 2:26(74),3:7 (20) | 0.9181 |
| Operation time (minutes) | 103 (91, 125) | 119 (99, 140) | 0.3155 |
| Bleeding (ml) | 24 (10, 50) | 10 (5, 38) | 0.3273 |
| Component design, CR, PS, n (%) | 25 (19), 111 (82) | 11 (31), 24 (69) | 0.1057 |
| Use of navigation, n (%) | 21 (15) | 5 (14) | 1.0 |
| Post-operative period (year) | 13 (12, 16) | 14 (13, 17) | 0.0956 |
| KSS total at 2012 | 112 (86. 132) | 108 (89, 129) | 0.8422 |

Note: Data are expressed as median and interquartile range (IQR)

Abbreviations: BMI, body mass index; GNRI, geriatric nutritional risk index; ASA, American Society of Anaesthesiologists, KSS, Knee society score

**Appendix Table 2**. Comparison of parameters between patients with GNRI <98 and control group in the second survey

| Parameters | **Control**  **(n=65)** | **GNRI <98**  **(n=10)** | ***p*-value** |
| --- | --- | --- | --- |
| Age at surgery (year) | 67 (61, 72) | 70 (53, 72) | 0.8270 |
| Sex, Male, Female, n (%) | 12 (18), 53 (82) | 1 (10), 9 (90) | 1.0 |
| BMI (kg/m^2^) | 27.4 (24.3, 30.5) | 21.1 (18.7, 22.6) | **<0.0001** |
| Preoperative albumin (g/dl) | 4.3 (4.1, 4.4) | 3.9 (3.6, 4.1) | **0.0004** |
| Preoperative GNRI score | 116.5 (108.3, 121.6) | 96 (94.6, 97.3) | **<0.0001** |
| ASA class1, 2, 3, n (%) | 1:3(5),2:54(83),3:8(12) | 1:0(0),2:7(70),3:3(30) | 0.2875 |
| Operation time (minutes) | 112 (92, 131) | 106 (88, 123) | 0.6336 |
| Bleeding (ml) | 21 (10, 42) | 33 (10, 53) | 0.4357 |
| Component design, CR, PS, n (%) | 14 (22), 51 (78) | 2 (10), 8 (80) | 1.0 |
| Use of navigation, n (%) | 10 (15) | 3 (30) | 0.3637 |
| Preoperative HKA angle (°) | 11.0 (4.5, 16.8) | 10.0 (0.5, 13.1) | 0.5456 |
| Postoperative HKA angle (°) | -1.0 (-3.1, 1.7) | 1.0 (-1.0, 1.7) | 0.2891 |
| Additional Surgery, n (%) | 9 (14) | 0 (0) | 0.5981 |
| Follow-up period (year) | 13 (12, 16) | 14 (13, 17) | 0.2066 |
| **KSS total at 2012** | 119 (90, 143) | 111 (82, 119) | 0.1365 |
| **KSS total at 2023** | 109 (84, 140) | 77 (49, 98) | **0.0101** |
| **Longitudinal change in KSS** | -4 (-20, 13) | -33 (-49, 8) | **0.0409** |

Note: Data are expressed as median and interquartile range (IQR)

Abbreviations: KSS, Knee society score; BMI, body mass index; GNRI, geriatric nutritional risk index; ASA, American Society of Anaesthesiologists; HKA,Hip-Knee-Ankle

**Appendix Table 3**. Descriptive data for the second survey cohort and patients excluded due to death or medical conditions

| Parameters | Control (n = 75) | Death or disability (n=36) | p-value |
| --- | --- | --- | --- |
| Age at surgery (year) | 68 (61, 72) | 70 (64, 75) | 0.0379 |
| Sex, Male, Female, n (%) | 13 (17), 62 (83) | 8 (22), 28 (78) | 0.6037 |
| BMI (kg/m^2^) | 26.9 (23.0, 29.8) | 24.5 (22.4, 26.9) | **0.0198** |
| Preoperative albumin (g/dl) | 4.2 (4.1, 4.4) | 4.1 (3.9, 4.4) | 0.1355 |
| Preoperative GNRI score | 114.1 (105.7, 120.5) | 108.6 (98.0, 116.3) | **0.0088** |
| Preoperative GNRI < 98, n (%) | 8 (11) | 14 (39) | **0.0009** |
| ASA class 1, 2, 3; n (%) | 1:3(4),2:61(81),3:11(15) | 1:2(6),2:22(61),3:12(33) | 0.0623 |
| Operation time (minutes) | 103 (89, 125) | 98 (83, 110) | 0.2015 |
| Bleeding (ml) | 34 (10, 55) | 21 (10, 35) | 0.1106 |
| Component design, CR, PS, n (%) | 16 (21), 59 (79) | 7 (19), 29 (81) | 1.0 |
| Use of navigation, n (%) | 13 (17) | 4 (11) | 0.5746 |
| Follow-up period (year) | 13 (11, 16) | 13 (12, 16) | 0.7101 |
| KSS total at 2012 | 116 (90. 141) | 103 (85, 123) | 0.1329 |

Note: Data are expressed as median and interquartile range (IQR)

Abbreviations: BMI, body mass index; GNRI, geriatric nutritional risk index; ASA, American Society of Anaesthesiologists, KSS, Knee society score
